# THE NEIGHBORHOOD CONTEXT AND ALL-CAUSE MORTALITY AMONG OLDER ADULTS IN PUERTO RICO

**DOI:** 10.1093/geroni/igac059.1936

**Published:** 2022-12-20

**Authors:** Catherine Garcia, Mary McEniry, Michael Crowe

**Affiliations:** Syracuse University, Syracuse, New York, United States; University of Wisconsin-Madison, Madison, Wisconsin, United States; University of Alabama-Birmingham, Birmingham, Alabama, United States

## Abstract

The neighborhood contexts in which older adults live are increasingly being recognized for their role in influencing disease processes and risk of death among the U.S. population. However, few studies have focused on neighborhood impacts among older populations residing in Puerto Rico– a U.S. territory –who are especially vulnerable to the effects of the environment as they “age in place” in the context of a budget crisis, the great recession, the debt crisis, and Hurricanes Irma and María. The combination of these events can obstruct access to neighborhood resources, services, and contexts considered necessary for promoting healthy aging. Thus, it is warranted to understand the effects of place on mortality in Puerto Rico, whose social and economic contexts differ from the U.S. and are more similar to that of other Latin American and Hispanic-Caribbean countries. We used 2000 U.S. Census data at the block-group level linked to the 2002 Puerto Rican Elderly Health Conditions Project with mortality follow-up to 2021 to examine neighborhood characteristics that are conceptualized as influencing mortality (e.g., residents without a high school degree; households receiving public assistance income; residents living below the poverty level; unemployed residents; residential stability; age structure). Multilevel mixed-effects parametric survival models with a Weibull distribution were estimated. Overall, results show that neighborhood socioeconomic disadvantage is associated with an increased risk of mortality among older Puerto Ricans. This suggests that older Puerto Ricans clustered in disadvantaged communities are more likely to experience a cumulative burden of social disadvantages that adversely impacts their longevity.

